# Experimental Sepsis Models: Advantages and Limitations

**DOI:** 10.5152/eurasianjmed.2023.23364

**Published:** 2023-12-01

**Authors:** Pelin Aydın, Huseyin Bekmez

**Affiliations:** 1 Department of Anesthesiology and Reanimation, Erzurum City Hospital, Erzurum, Turkey; 2 Department of Pharmacology, Atatürk University Faculty of Medicine, Erzurum, Turkey

**Keywords:** Sepsis, experimental model, CLP, LPS

## Abstract

Sepsis is a major health problem that causes millions of deaths worldwide every year. Due to the complexity of its pathophysiology, there is no clear treatment method for it. Existing treatments impose an additional financial burden on the health systems of countries every year. Clinical and preclinical studies are continuously being conducted in order to prevent the development of sepsis, treat patients with sepsis, reduce mortality, and solve the socioeconomic problems that arise from it. However, it is not possible to directly test every study and potential new treatment in humans. Preclinical studies enable an understanding of pathophysiological events and the development of targeted therapies. For this purpose, many experimental sepsis models have been and continue to be applied. The extent to which these models can reflect the human sepsis condition is an important issue that needs to be emphasized. Each method has different strengths and weaknesses. Researchers should choose the most appropriate experimental model according to the characteristics of the experiments they plan and, if possible, conduct their studies on different models.

**Main Points**

- Researchers frequently apply experimental sepsis models to explain the pathophysiology of sepsis, understand the function of pathways, and demonstrate the efficacy of new therapeutic strategies.
- These different aspects of the models prevent one from being more prominent than the others.
- Researchers should choose the most appropriate experimental model according to the characteristics of their experiment.

## Introduction

Sepsis is a condition resulting from a dysregulated inflammatory response to infection, leading to serious clinical consequences.^1,2^ Due to an infection or traumatic injury in an organism, the immune system is activated and many cytokines are released.^3^ The result is a dysregulated inflammatory response, hemostatic changes, multiorgan dysfunction, and ultimately, death.^4^ When global data were analyzed, it was seen that sepsis is one of the most common causes of death in intensive care units (ICUs). A mortality rate of 30% was also mentioned.^5^ At the same time, sepsis causes serious socioeconomic problems. When data on the costs of hospitalized patients in the United States were analyzed, sepsis was the most costly condition.^6^ However, there is no clear treatment for this important health problem to date. For this purpose, many clinical and experimental studies are being conducted. New treatment methods and pharmacological agents are being tested by creating experimental sepsis models.^7,8^ Preclinical experimental models have a crucial role in the development of new treatment strategies for the disease. Many different experimental models have been used to generate sepsis. Each of these methods has advantages and disadvantages.^9^ The important point here is that the method to be used should be able to reflect human sepsis in the best possible way. Clinical definitions of sepsis and recommended treatments are being updated every day, and necessary adjustments are being made. Sepsis, which has been known since the time of Hippocrates, was called systemic inflammatory response syndrome in 1991, and then the definitions of sepsis clinically were reorganized in 2016, and a common consensus was reached. A reorganization was made in 2021.^10,11^ However, until 2017, there was no systematic review of preclinical sepsis models, and no clear guidelines were prepared. The limitations of the preclinical studies were identified, and standardization was attempted. However, this consensus did not receive official approval from professional organizations.^12^

This review will highlight the potential strengths and weaknesses of commonly used experimental sepsis models and provide guidance for researchers to select the appropriate sepsis model for their experiments.

## Experimental Models

### Models of Peritonitis

#### Cecal Ligation and Puncture Method

For decades, the cecal ligation and puncture (CLP) model has been the most widely used animal model to study the pathogenesis of human sepsis. Because it closely resembles the characteristics of human sepsis, it has become the mainstay of sepsis research for many researchers. At the same time, this is also one of the reasons why researchers prefer it, as it provides insight into a better understanding of sepsis physiology and investigates new signaling pathways in a therapeutic direction.^13-15^

In this sepsis model, animals are appropriately anesthetized. After achieving an adequate depth of anesthesia, the midline of the abdomen is sterilized, and a laparotomy is performed. The cecum is freed, ligated below the ileocecal valve, and then punctured with a sterile needle. A small amount of feces is allowed to come out by gently squeezing the puncture site. Then, it is placed back into the abdominal cavity. The abdomen is closed properly.^16,17^ The aim of the CLP method is to puncture the cecal barrier, leading to peritoneal infection. Perforation of the cecum and exposure of the intestinal contents leads to bacterial contamination, which causes bacterial peritonitis. This is followed by bacteremia, activation of the inflammatory response, multiorgan dysfunction, and finally, death.^18^ Since it is peritonitis due to contamination, it is similar to clinical conditions such as perforated appendicitis or diverticulitis.^19^ This model induces the same immune response as in human sepsis. The resulting hemodynamic, inflammatory, and biochemical changes are very similar to those of human sepsis. And, as in human sepsis, there is a more gradual increase in plasma cytokines. However, this increase is quite consistent.^13,20,21^ The severity of the resulting sepsis varies depending on the length of the cecal area ligated, the size of the needle, and the number of punctures.^13^ The main advantage of the model is its simplicity. It is completed with a simple surgical procedure.^19^ The second important advantage, as mentioned above, is that it is mechanistically very similar to human sepsis. However, it has been observed that the protocols applied in different laboratories are quite different from each other.^22^ The reasons for this variability include the type and dose of anesthetic agent, the surgical skill of the practitioner, the length of the ligated portion of the cecum, the size and number of holes in the cecum, the amount of feces that is scooped out of the cecum, whether antibiotics should be given, and how fluid resuscitation should be performed.^23^ Due to these variables, it is very difficult to ensure standardization.^24^ In addition, ischemia/necrosis of the intestine and contamination of the peritoneum by bacteria are necessary for sepsis to fully develop. Without these conditions, organ failure and death do not develop in the animals, and a complete sepsis profile cannot be created.^25^

#### Cecal Slurry Model

Cecal slurry (CS) is a different model of intraabdominal sepsis. This model is based on the intraperitoneal injection of a defined and characterized amount of cecal contents. It is a model designed to reflect the human neonatal condition of necrotizing enterocolitis, which is usually seen in premature newborns and has a high mortality rate. Researchers studying neonatal sepsis often prefer this model. In fact, many neonatal sepsis researchers consider it the gold standard. The small body size of newborn mice makes it difficult to perform the CLP procedure. At the same time, CLP is not preferred because rodent mothers have a tendency toward cannibalization after the surgical procedure.^26,27^ Unlike the CLP method, the CS method does not involve a surgical procedure. Therefore, there is no surgical tissue trauma and no necrotized tissue. Another important difference is that it causes a stronger but shorter early inflammatory response.^22,28^

#### Colon Ascendens Stent Peritonitis

Colon ascendens stent peritonitis, like CLP, is a surgical procedure. In this model, a stent is placed in the ascending colon between the intestinal lumen and the abdominal cavity.^29^ It has been reported that it mimics the pathophysiological changes in human sepsis better than the CLP model.^30^ However, the procedure is more difficult than the CLP model. It has also been observed that in the immune response to sepsis, pro-inflammatory and anti-inflammatory cytokine responses occur almost simultaneously.^31^

#### Cecal Ligation and Incision

Cecal ligation and incision (CLI) is a surgical procedure designed to obtain a severe sepsis model that can meet international sepsis criteria. The CLI creates a more acute-onset sepsis model compared to the CLP model. Since the immune, metabolic, and hemodynamic responses generated by this model have not been sufficiently characterized, it is not yet as widely used as the CLP model.^32^

The advantages and limitations of experimental sepsis models are summarized in Table 1.

### Toxin Models

#### Lipopolysaccharide Application

Lipopolysaccharide (LPS) is the main component of the cell wall of gram-negative bacteria and is often preferred to create an experimental sepsis model. It can be administered to experimental animals via intravenous or intraperitoneal injection.^33^ In the LPS sepsis model, no active infection occurs because the experimental animal is directly administered endotoxin, i.e., no live bacteria. Although an active infection does not occur, the immune system is activated in the subject, and a process is initiated in which we can obtain information about the main pathways in the pathophysiology of sepsis. However, the difference here is that the hypodynamic phase of sepsis occurs without the hyperdynamic phase. LPS administration is a controlled, reproducible model. Lipopolysaccharide cannot fully represent host–pathogen interactions or the development of polymicrobial sepsis because the immune system does not necessarily eliminate the pathogen.^34^ Lipopolysaccharide represents pathogen-associated patterns of gram-negative bacteria. In order to initiate the immune response, LPS first interacts with and activates the specific immune cell receptor, toll-like receptor 4 (TLR 4).^35^ It then initiates the immune response by activating many intracellular signaling pathways, particularly the nuclear factor kappa B (NF-κB) signaling pathway. In experimental studies, the immune response to LPS and modulation of intracellular signaling pathways have been shown in detail.^36,37^ The degree of the desired immune response can often be determined by varying the dose of LPS or by using LPS types with different biological activities. It is important to note that, in this model, LPS sensitivity varies greatly from species to species. The process of sepsis in humans shows a slow, progressive course over days. Following LPS injection, there is a very strong increase of proinflammatory cytokines in the plasma within a very short period of time, which more rapidly become soluble.^38^ Another issue that needs to be emphasized here is that LPS is specific to gram-negative bacteria and since the toxin is administered directly to the organism, gram-positive microorganisms and host–pathogen interactions in polymicrobial sepsis are ignored. For this reason, it has been argued that the direct administration of determined amounts of LPS to the subjects is not a real sepsis condition but can be considered an intoxication model.^33^

### Gram-Positive Toxins

This is based on the administration of toxins such as peptidoglycan and lipoteichoic acid (LTA) of gram-positive pathogens. The interest in LPS is quite high due to its fundamental role in toxin-induced sepsis models. However, *S. aureus* is one of the most frequently isolated bacteria from patients with sepsis, and with the increasing incidence of resistant pathogens such as methicillin-resistant *S. aureus*, there has been a trend towards a better understanding of gram-positive sepsis.^39^ It should also be kept in mind that newborns are more susceptible to gram-positive infection.^40^ These gram-positive toxins, like LPS, are cell wall components that act as pathogen-associated molecular pattern molecules (PAMPs) and are known to be associated with toxemia. Injecting peptidoglycan and LTA into subjects causes many symptoms similar to those seen in LPS endotoxemia. However, it has been found that even if some signaling pathways are common, the main pathways may differ. The most important difference is the recognition receptor type. Nucleotide oligomerization domain (NOD) receptors are the main receptors for peptidoglycan. Nucleotide oligomerization domains recognize PAMPs through repeats rich in the amino acid leucine, just like TLRs. The most important difference between NOD receptors and TLRs is their location in the cell. Toll-like receptors are located on the cell membrane, while NOD receptors are located in the cytosol.^41^ Lipoteichoic acid is an endotoxin known to activate the innate immune system and activate TLR2.^42^ In a study in which LTA was administered in the same amount as LPS, it was reported to cause a lower amount of cytokine release. However, the condition created by LTA was found to be sufficient for the formation of a septic picture.^43^

### Application of Live Pathogens

This is a model in which live bacteria is administered to an organism via various routes, most commonly intraperitoneally or intravenously. Among gram-negative microorganisms, *Escherichia coli*, Bacteroides fragilis, *K. pneumoniae*, *A. baumannii*, and *P. aeruginosa* are used. Among gram-positive pathogens, *S. aureus* and *S. pneumoniae* are preferred.^38^ In this model, there is no focus on infection, where microorganisms multiply and spread. Therefore, it cannot be said to create a complete sepsis profile.^44^ However, in the process of sepsis, there is a septic focus in the body of the patient, and there is a continuous release of bacteria from this focus. Some researchers argue that the model is not appropriate for this reason.^9^ Human sepsis is polymicrobial. In this model, a single type of pathogen is usually administered. Pathogens are administered to the subjects in large quantities. Perhaps the most powerful aspect of the model is that it provides septic conditions specific to the site of administration. It can mimic peritonitis when administered intraperitoneally, catheter-mediated infection when administered intravenously, or conditions such as meningococcemia and urosepsis.^45,46^ An important issue to be emphasized here is that host and bacterial characteristics exhibit species-specific behaviors in infectious situations. Some pathogens may cause systemic infections in rodents but not in humans.^47^

Sepsis due to fungal infections is frequently seen in immunocompromised patients and especially in ICUs.^48^ Therefore, it would be wrong to talk only about bacteria in this sepsis model, as there are also various fungal applications of this model. A sepsis model in mice has been described using *Candida auris*, which is among the very serious nosocomial pathogens.^49^ Again, a sepsis model was created in mice by the intravenous administration of *Candida albicans*. Severe sepsis was observed, and renal failure was shown to develop.^50^

The lack of an active bacterial focus in live pathogen applications and the need for an accurate reflection of the septic picture have led researchers to develop a new method in which bacteria are added to fibrin clots, which are implanted into the peritoneal cavity. The clot acts as a focus of infection, allowing the continuous release of bacteria.^51^ The severity of the sepsis can be regulated by changing the bacterial concentration and clot density. The most important disadvantage of the method is that it causes monoinfection. In this respect, it lags behind the polymicrobial development of true sepsis.^52^

## Conclusion

It seems that the current experimental models need to be further optimized to better adapt to human sepsis. The strengths and weaknesses of each of the current experimental models have been described in detail. These different aspects of the models prevent one from being more prominent than the others. Of course, they play a very important role in explaining the pathophysiology of sepsis, understanding the functions of pathways, and demonstrating the effectiveness of new treatment strategies. At the same time, however, the extent to which these models can truly reflect the sepsis that occurs in the clinic is a matter of debate. The most important point here is that researchers should choose the most appropriate experimental model according to the characteristics of their experiments. Perhaps, to increase the success rate, it may be recommended to conduct studies on different models at the same time, if possible.

## Figures and Tables

**Table 1.** Comparative Characteristics of Experimental Sepsis Models

Model	Advantages	Limitation
CLP	1.Simple surgical procedure. 2. Exhibits very similar features to the clinical sepsis model. 3. Tissue ischemia develops. It also mimics the development of polymicrobial peritonitis. 4. Sample preparation is not required. 5. Both proinflammatory and anti-inflammatory immune responses are activated. 6. The severity of sepsis can be regulated by changing the number of holes, hole diameter, or length of the ligation site.	1. Failure of the surgical procedure. 2. Tissue trauma occurs. 3. Variability between laboratories. 4. Gender and age variability. 5. Inapplicability in newborns. 6. Reproducibility is poor because standardization is difficult to achieve. 6. It is difficult to control the bacterial load in the fecal contents leaking into the peritoneum and the magnitude of the challenge of sepsis.
CS	1. It does not require a surgical procedure. 2. The model is very simple and reproducible. 3. The model mimics polymicrobial peritonitis. 4. The model is the gold standard for neonatal sepsis. 5. The dynamics of sepsis can be regulated by changing the amount of injected slurry or feces.	1. The model is difficult to standardize (Due to differences in microbiota composition and sample preparation). 2. The metabolic, hemodynamic, and immunologic features of clinical sepsis may not always be fully mimicked.
CASP	1. The model is a surgical procedure. 2. The model causes diffuse peritonitis with a systemic infection. 3. The course of sepsis development is regulated by changing the stent diameter or removing the stent. 4. Microbiota diversity is largely preserved. 5. Abscess does not form.	1. Surgical procedure is difficult. 2. It requires special surgical technique. 3. Tissue trauma occurs. 4. Not for use in newborns. 5. In the model, hemodynamic, immunological, and metabolic changes are less characterized than in CLP. 6. Less experience
CLI	1. Designed to model severe sepsis. 2. It creates an acute onset sepsis model.	1. Surgical procedure is difficult. 2. In the model, hemodynamic, immunological, and metabolic changes are less characterized than in CLP. 3. Less experience.
LPS	1. It does not require a surgical procedure. 2. LPS is an easy model to implement. 3. The model is the toxicosis model. 4. Standardized and reproducible. 5. LPS can be applied in several ways. 6. It models the acute phase of Gram-negative sepsis well. 7. The course of sepsis development can be regulated by altering the amount or biological activity of LPS.	1. Since a single toxin is administered, it may not completely mimic the polymicrobial sepsis and responses in human sepsis. 2. Active infection does not occur. 3. Responses to the toxin vary within and between species. 4. Represents an acute but short-lived immune response. 5. LPS doses, route of administration, and rate of administration can affect host responses to LPS. Therefore, variable hemodynamic responses may be seen.
Live pathogens	1. The model is easy, less invasive, and reproducible. 2. Intravenous infusion of live organisms is suitable for studying the kinetics of clearance of microorganisms from the blood. 3. Bacteria can be administered in many ways such as intravenously, intraperitoneally, intranasally. 4. It allows the use of pathogenic bacterial strains that are relevant to the body compartment. 5. The course of sepsis is regulated by changing the number of bacteria to be administered.	1. Model uses a single bacterial species, whereas clinical sepsis is polymicrobial. 2. For this method, the bacteria need to be reproduced and quantified 3. Variability between laboratories may exist in practice. 4. Endotoxemia may develop if the bacterial load is excessive in practice. 5. The host response varies depending on the type and quantity of bacteria, route, and duration of administration. 6. The hemodynamic, immunological, and metabolic features of clinical sepsis have not been adequately modeled.

CLP, cecal ligation and puncture; CS, cecal slurry; CASP, colon ascendens stent peritonitis; CLI, cecal ligation and incision; LPS, lipopolysaccharide.
